# Optimized Expression of Full-Length IgG1 Antibody in a Common *E. coli* Strain

**DOI:** 10.1371/journal.pone.0010261

**Published:** 2010-04-20

**Authors:** Conrad En Zuo Chan, Angeline Pei Chiew Lim, Annie Hoi Yi Chan, Paul A. MacAry, Brendon John Hanson

**Affiliations:** 1 Medical Countermeasures (Biological) Laboratory, Defense Medical and Environmental Research Institute, DSO National Laboratories, Singapore, Singapore; 2 Immunology Program, Department of Microbiology, National University of Singapore, Singapore, Singapore; University of Crete, Greece

## Abstract

Multi-polypeptide proteins such as antibodies are difficult to express in prokaryotic systems such as *E. coli* due to the complexity of protein folding plus secretion. Thus far, proprietary strains or fermenter cultures have been required for appreciable yields. Previous studies have shown that expression of heterologous proteins in *E. coli* can be enhanced by the reduction of protein translation rates. In this paper, we demonstrate that useful quantities of full-length IgG can be expressed and purified from the common *E. coli* laboratory strain HB2151 in standard shaking culture using a simple strategy of reduced inducer concentration combined with delayed induction times to modulate translation rates. Purified IgG had only marginally reduced avidity compared to mammalian derived IgG. This indicates that this technique can be used to derive antibodies of potentially equal utility as those expressed in mammalian cell culture, particularly for applications where effector functions mediated by the glycosylated residues in the Fragment Crystallizable (Fc) portion of the immunoglobulin are not required.

## Introduction

In the immune system and also for many therapeutic antibody applications, the Fc region recruits receptors and cell types that maintain the circulating half life of unbound antibodies. With antibody-antigen interaction, the Fc region initiates the main antibody effector functions: complement-dependent cytotoxicity (CDC), antibody-dependent cellular cytotoxicity (ADCC), and phagocytosis, which ultimately result in clearance of the antigen. For many therapeutic applications, although retention of the circulating half life of the antibody is crucial, recruitment of effector functions is not necessary.

Traditionally, full-length antibodies have been expressed in mammalian tissue culture, primarily because the motifs within the Fc region responsible for effector ligand recruitment require the presence of both specific amino acids as well as glycosylation [1], [2], [3] Indeed, alteration of the glycoform can affect the affinity of the Fc for various receptor domains and hence determine the specific type of effector function activated [4], [5], [6], [7]. In the case of antibody circulating half life, the motif within the Fc region responsible for receptor interaction is not dependant on glycosylation, and expression of aglycosylated antibodies does not affect circulating half life [3], [8].While production of aglycosylated antibodies can be achieved in mammalian cell expression through deletion of the glycosylation signal, recently, aglycosylated antibodies have been produced via expression in *E. coli* [8], [9]. However, removal of periplasmic proteases via molecular engineering of the *E.coli* strain used, along with fermentation culture, was required to achieve appreciable yield.

Antibodies are not ideal for expression in *E. coli* as they are complicated multimeric proteins made from two different polypeptides, the heavy (HC) and light chains (LC), which must be exported into the periplasm, folded properly and form the appropriate disulfide bonds. As such, considerable effort has been made to optimize bacterial hosts for antibody and antibody fragment expression. Engineering of oxidizing cellular environments, co-expression of molecular chaperones, use of periplasmic protease deficient strain of *E. coli* and balancing of heavy and light chain expression have all enabled increased yield of up to 1 mg/L [8], [10], [11]. However, these options often require some degree of further optimization such as balancing expression of each polypeptide chain, or the use of proprietary modified *E. coli* strains which are not readily available. Modification of translation initiation regions (TIRs) to reduce protein translation rates has also had some success at improving overall yield [12]. The lower translation rate is believed to decrease protein load on the secretory system, reducing the accumulation of unprocessed products in the cytoplasm. Indeed, the high level expression of antibody obtained in fermentor cultures was obtained using balanced but low strength TIRs for both heavy and light chains [8].

In this study, we explored strategies for optimization of antibody expression in general laboratory strains of *E. coli* using simple methods for reducing translation rates. These include the use of a low-copy number plasmid, reduction of inducer concentration and induction of antibody HC/LC at late log phase. Single step purification on Protein A resulted in co-elution of bacterial proteins along with degraded heavy chain. Introduction of a second purification step with Protein L successfully removed contaminating proteins and heavy chain fragments.

## Results

### Preliminary bacterial IgG expression

**Figure 1:**
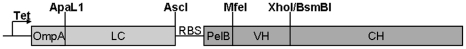
Construction of tet promoter bacterial IgG expression plasmid. pASK-IBA2 plasmid using a *tet* promoter was used as the backbone expression vector. The appropriate restriction sites for cloning in of the light and variable heavy chains, leader sequences (OmpA, PelB) and the constant heavy chain sequence (CH) were added. Light (LC) and variable chains (VH) were cloned in as a complete construct together with the intercistronic ribosomal binding site (RBS) from the phage display vector or as separate constructs from the 4G2 mammalian IgG expression vector.

**Figure 2:**
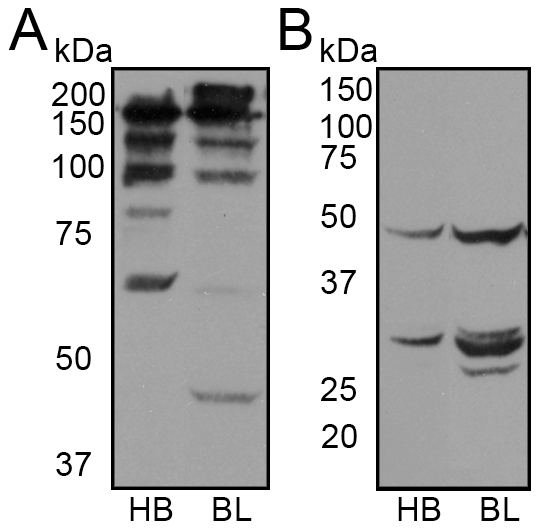
Initial bacterial IgG expression and periplasmic extraction. (A and B) Non-reducing and reducing immunoblot of periplasmic extract from overnight expression of 4G2 in HB2151 (HB) and BL21(DE3) (BL) *E. coli* strains, respectively. All blots were probed with anti-Human Fc-HRPO.

For expression of full length IgG we constructed a bicistronic expression cassette driven by a tetracycline inducible promoter where the light chain contained an OmpA leader sequence and the heavy chain contained a PelB leader sequence separated by an intercistronic ribosomal binding site (Figure 1). Using standard conditions, our initial attempts to produce full-length IgG in *E. coli* resulted in undetectable yields of fully assembled IgG and only unassembled or extensively degraded heavy chain fragments were detected on immunoblot (data not shown). In order to reduce the degradation of IgG, we utilized the *E. coli* strain commonly used for protein expression, BL21(DE3), which is deficient in the cellular proteases Lon and OmpT. Periplasmic extraction was also employed rather than whole cell lysis, in the hope that only fully assembled IgG would be extracted and not unprocessed heavy and light chain. Analysis of the periplasmic extract after overnight expression of a chimeric antibody termed 4G2, showed fully assembled IgG with both HB2151 and BL21(DE3) (Figure 2A). Surprisingly, reduction of the extracts to allow resolution of the heavy and light chains revealed the presence of increased degradation of the heavy chain with the protease deficient BL21(DE3) compared to HB2151, suggesting that proteolysis of the heavy chain is not due to Lon or OmpT proteases (Figure 2B).

### Optimization of expression in small scale culture

Expression of full-length IgG, Fab as well as other proteins appear to be significantly enhanced by reduction of protein translation rates and ten-fold reduction in translation rates has been shown to significantly increase secretion of heterologous proteins in *E. coli* [12]. We therefore explored various methods for reducing translation rates to investigate if this improved yield. A simple method for adjusting these rates is to reduce inducer concentrations and such modulation has been previously demonstrated in similar tetracycline promoter based systems in other bacterial hosts such as *Mycobacteria* as well as *E. coli*. [13], [14] Vectors that employ a tetracycline based promoter also enable tight control of expression that is independent of the host strain or metabolism [15], and dictated our initial construction of the bacterial IgG cassette in this vector Previous experiments using the Tet repressor had shown that below 50 ng/ml, concentration of inducer becomes limiting although the relationship between expression yield and inducer is not linear [13], [14]. We therefore carried out an induction at the manufacturer's recommended concentration of 200 ng/ml, and at 50 ng/ml and 20 ng/ml.

Other methods which can reduce translation rates include induction of expression when the bacteria are approaching late log phase. This was used previously by Mazor et al for the production of full-length IgG [9]. Accordingly, aside from the standard OD_600_ 0.6 for induction we also tested expression when induction was started with culture at OD_600_ 1.0. Use of low copy number plasmids has also been shown to decrease translation rate [14]. In the course of constructing our expression vector, we generated both a low copy and high copy version of our expression plasmid, by addition of a single base pair mutation in the origin of replication. The difference in copy number between the high and low copy plasmids, based on miniprep DNA yields, is estimated to be approximately eight- to ten- fold.

**Figure 3:**
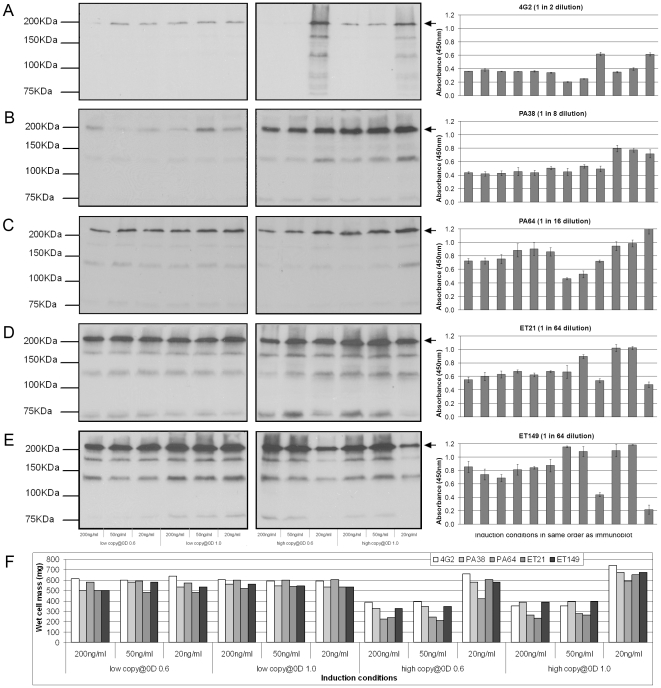
Small scale optimization of bacterial IgG expression. (A-E) Left and middle panels: Non-reducing immunoblot of 7.5 µl clarified cell lysate from small scale overnight expression of bacterial IgG in shaking culture. All blots were probed with anti-Human Fc-HRPO and adjusted to ensure equal intensity. Arrows indicate fully-assembled IgG. Right panel: Direct binding ELISA indicating levels of functional IgG. Background binding signal was negligible for all cell lysate samples at the indicated dilution or neat. Error bars were calculated from average of three or four observations. Expressed antibodies were 4G2 (A), PA38 (B), PA64 (C), ET21 (D), ET149 (E). (F) Variation in wet cell mass for all five antibodies under different induction conditions. Mass is indicated in mg

To analyze expression induced by our new construct, we employed five different full length IgGs. One was a chimeric antibody derived from the anti-dengue mouse monoclonal hybridoma 4G2; and the other four fully human antibodies isolated from a naïve human phage display library; of which two (ET21 and ET149) were raised against *Clostridium perfringens* epsilon toxin and the other two (PA38 and PA64) against *Bacillus anthracis* protective antigen. Studies were carried out in small scale shake cultures with various combinations of different concentrations of inducer, induction at different optical densities (OD) and using either high or low copy expression vector, as indicated. In order to prevent leakage of expressed antibody from the periplasm during growth by mechanical sheer, which commonly occurs during periplasmic expression in the HB2151 *E. coli* strain, protein expression was carried out in non-baffled flasks at a slower shaking speed of 120 rpm. Levels of fully assembled and functional IgG in the clarified cell lysate were then determined by immunoblot, standard induction conditions (200 ng/ml inducer, high copy plasmid & induction at OD_600_ 1.0) was also used as a comparison between immunoblots, and direct binding ELISA respectively (Figure 3).

Initial experiments indicated that variation of inducer concentration resulted in significant differences in wet cell mass (Figure 3F), which can have a significant influence on overall yield. Therefore, to distinguish whether improved yield is due to an increase in wet cell mass or an increase in levels of fully assembled IgG per cell (unit yield), pellets were weighed after harvesting and resuspended in a volume of lysis buffer adjusted for wet cell mass thus ensuring that subsequently derived lysate samples are from equivalent wet cell masses.

The levels of fully assembled and functional IgG as measured by immunoblot and direct binding ELISA respectively correlate well for all five antibodies expressed. In general, the strongest full sized IgG band (≈200 kDa) on the immunoblot and the highest signal by ELISA were obtained when the same set of conditions were used (Figure 3A-E). Furthermore, there was a significant increase in wet cell mass when the lowest inducer concentration (20 ng/ml) was used in conjunction with high copy plasmid with up to approximately two-fold increase over the standard induction conditions or other conditions with higher inducer concentrations (Figure 3F). When low copy plasmid was used a smaller gain in wet cell mass was observed over standard induction conditions, which was unaffected by changing induction OD and inducer concentration. These effects were observed for all five antibodies tested and consistently the largest wet cell mass yield was observed with the combination of high copy plasmid, 20 ng/ml inducer concentration and induction at OD_600_ 1.0. However, this combination did not always give the best unit yield.

For chimeric 4G2 and PA64, this set of conditions was indeed the best as indicated by both immunoblot and ELISA, although for 4G2 induction with 20 ng/ml inducer at OD_600_ 0.6 showed a larger full sized IgG band by immunoblot; however the ELISA signal did not increase (Figure 3A & C). For PA38 any inducer concentration in combination with high copy plasmid and induction at OD_600_ 1.0 gave the highest yields (Figure 3B). In contrast, for ET21 and ET149 the highest unit yields were obtained using the higher inducer concentrations of 200 ng/ml or 50 ng/ml in combination with a high copy plasmid and induction at OD_600_ 1.0 (Figure 3D & E). Interestingly, when low copy plasmid was used, unit yields were less affected by variation of OD or inducer concentration (Figure 3A-E). In general, with the exception of ET149, use of low copy plasmids gave equivalent or better unit yields over standard induction conditions (200 ng/ml inducer at OD_600_ 0.6); however the optimized conditions with the high copy number plasmid always showed the greatest unit yield.

Degradation was observed to some extent for all five antibodies although the smaller sized degradation fragment bands were fainter than the full sized bands, indicating that it was not a significant problem overall (Figure 3A-E).

### Comparison of yield by larger scale expression

Based on the analysis of cell lysate from the small scale expression of five antibodies, we have determined that best expression levels can be obtained using only two sets of conditions: for PA38, PA64 and 4G2, optimal expression was observed from the high copy plasmid, induced at OD_600_ 1.0 with 20 ng/ml inducer. The yield would also be enhanced further by the greater wet cell mass obtained under this condition. For ET21 and ET149, 50 ng/ml inducer appears to be optimal in comparison to 20 ng/ml (with high copy plasmid and induction at OD_600_ 1.0) but due to the reduced wet cell mass overall yield might not be better. In order to determine which of the two combinations above gave the best overall yield, a larger scale expression was carried out with a representative antibody which showed optimal expression with each of the two combinations; 4G2 for 20 ng/ml inducer and ET149 for 50 ng/ml inducer.

**Figure 4:**
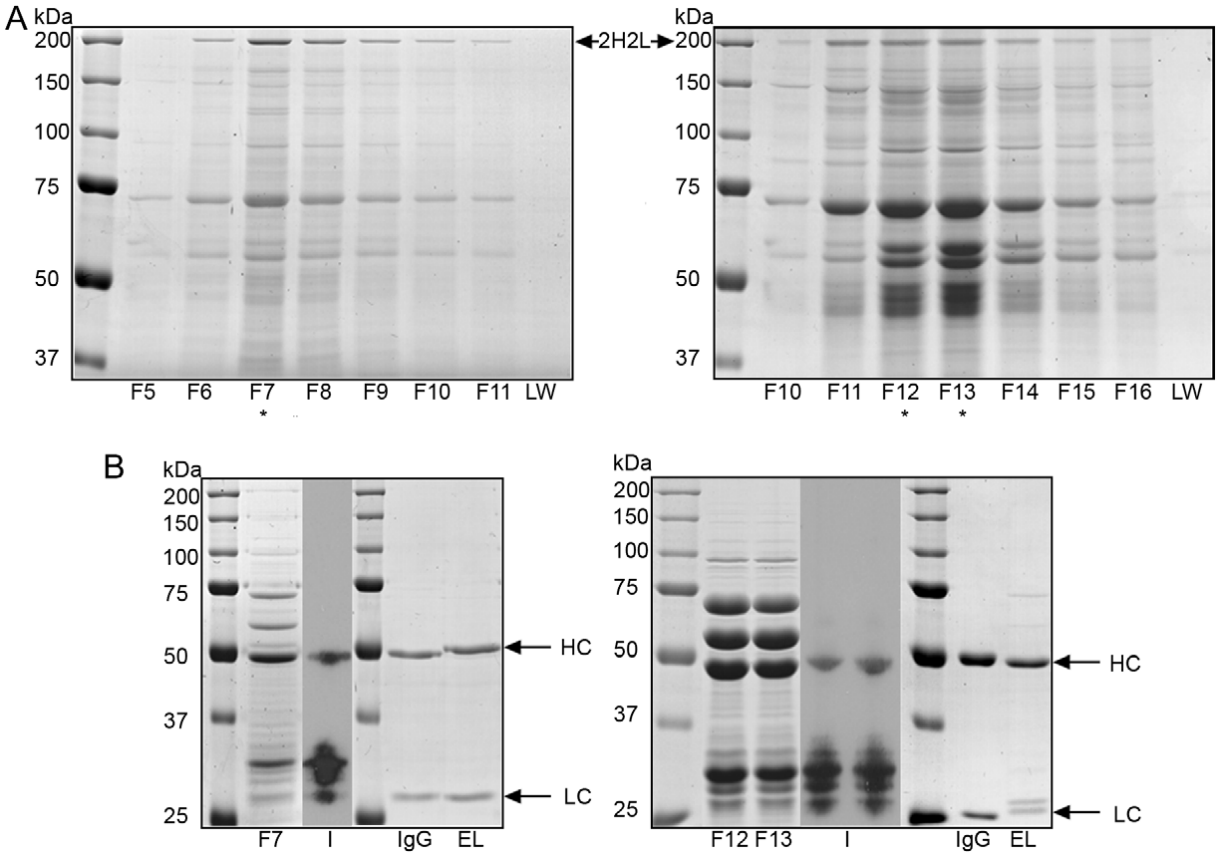
Large scale purification of bacterial IgG. (A) Non-reducing coomassie gel of peak fractions from Protein A HPLC purification of cell lysate. Left panel: 4G2 fractions 5 to 11 (F5 to F11). Right panel ET149 fractions 10 to 16 (F10 to F16). 30 µl of each fraction was run on the gel. A sample of the wash (LW) was run and shows no contaminants present indicating the column was sufficiently washed to remove non-binding proteins. Fully assembled IgGs (2H2L) are indicated. (B). Reducing coomassie gels (4G2-F7, ET149-F12 & 13) and adjacent immunoblots (I) showing representative fractions from each Protein A elution (indicated with * on panel A). 30 µl of each fraction was loaded for coomassie and 3.75 µl for immunoblot. Blots were probed with both anti-IgG Fc and anti-Kappa chain polyclonals showing that majority of protein bands in the fraction are neither IgG heavy chain nor light chain. A separate reducing coomassie gel shows 2 µg of bacterial IgG pooled eluate (EL) after Protein L purification showing successful removal of the contaminating proteins and degraded heavy chain fragments. The equivalent amount of mammalian cell culture-derived IgG (IgG) was loaded for comparison. Individual heavy (HC) and light (LC) chains are indicated although the light chain for ET149 appears as two separate species.

Purification was carried out on a 1 ml Protein A HPLC column and elution of IgG was tracked using absorbance at 280 nm. However, analysis of the individual peak fractions by SDS-PAGE revealed the presence of multiple protein bands (Figure 4A & B). Analysis of selected fractions by immunoblot (Figure 4B) revealed that some of these protein bands were not antibody heavy or light chains as they were not detected with antibodies against either human Fc or kappa light chain. This indicated that they are more likely to be bacterial proteins that co-purified with the bacterial expressed IgG. The non-specific elution of bacterial proteins was unexpected, as previous papers had reported clean elution of bacterially expressed full-length IgG from Protein A [8], [9]. The decoration of protein bands between the heavy and light chain on the reducing immunoblot indicates possible degradation of the heavy chain (Figure 4B).

Due to the impurity of the Protein A eluate, it was decided to carry out an additional purification step using Protein L, which binds the antibody kappa light chain. Sequential purification first on Protein A followed by Protein L enabled isolation of antibodies that contain both heavy and light chain, thus eliminating any unpaired heavy or light chain along with degraded fragments and contaminating bacterial proteins (Figure 4B).

Comparison of overall yields from 1.6L of culture by quantitation of total protein in pooled Protein L eluate from the two different conditions after sequential protein A and protein L purification indicated that as expected, expression of 4G2 at OD_600_ 1.0 using an inducer concentration of 20 ng/ml and high copy plasmid resulted in yields with approximately two-fold improvement (12.6 µg vs. 6.15 µg). However, for ET149 a change of inducer concentration to 50 ng/ml gave only slightly improved yields (6.3 µg vs. 6.15 µg), demonstrating the compensating effect of greater wet cell mass at 20 ng/ml inducer concentration.

### Comparison of bacterially expressed IgG with mammalian expressed IgG

**Figure 5:**
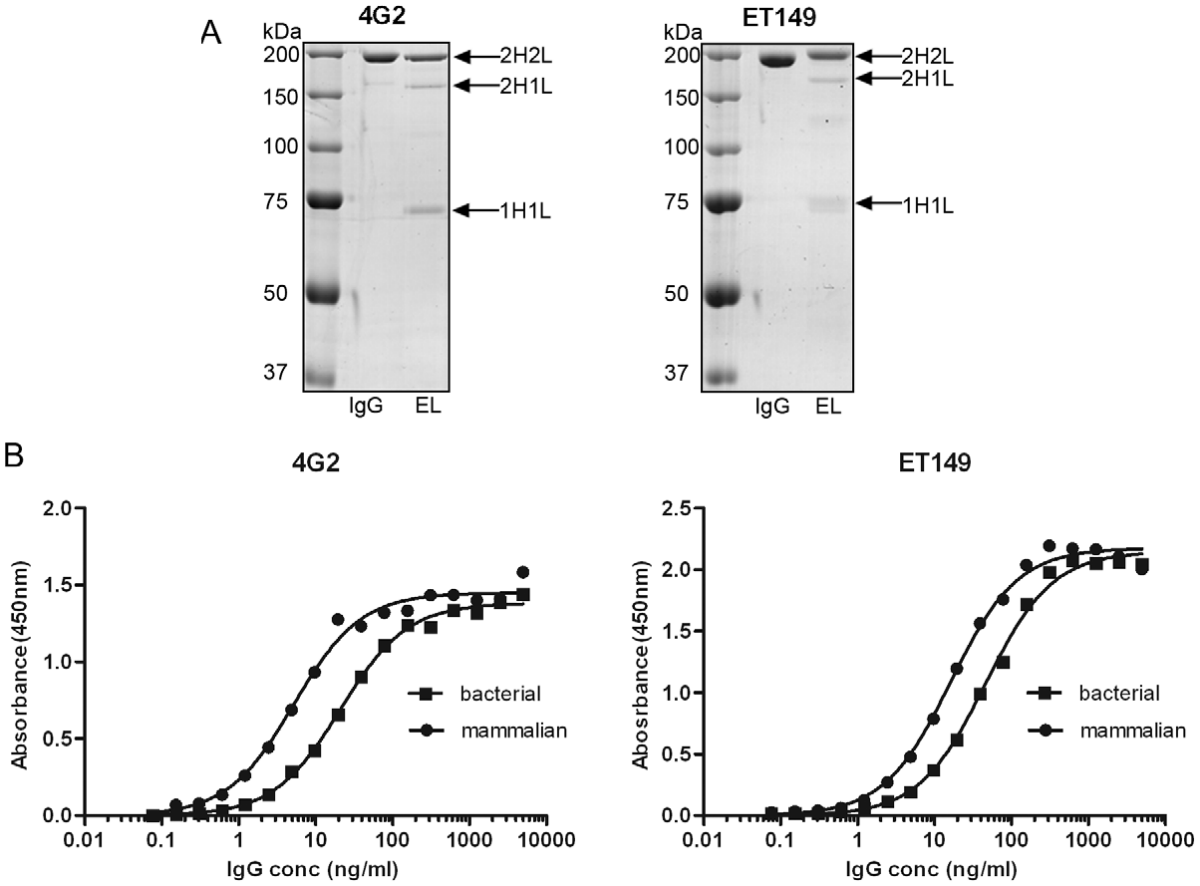
Comparison between bacterial and mammalian IgG. (A). Non-reducing SDS-PAGE of 2 µg of pooled bacterial IgG eluate after protein L purification (EL) and mammalian cell culture-derived IgG (IgG) indicates that the majority of purified IgG was fully assembled IgG (2H2L) although partially assembled IgG (2H1L, 1H1L) are also present. (B). Direct binding ELISA of serially diluted bacterial (-▪-)- and mammalian cell culture (-•-)-derived IgG against Dengue serotype-2 virus and epsilon toxin showing similar binding curves. Binding of antibody was detected using anti-Human IgG Fc-HRPO as the secondary antibody.

Following removal of the bacterial contaminant proteins and heavy chain degradation products through protein L purification we next analyzed the presence of fully assembled tetrameric IgG. Analysis of the protein L eluate on non-reducing SDS-PAGE indicated that a large proportion of the purified chimeric 4G2 and ET149 IgG was present as fully assembled IgG (Figure 5A). However partially assembled IgG representing 2HC-1LC and 1HC-1LC was also evident. The increase of partially assembled IgG seen with expression in *E. coli* could be due to inefficient disulfide bond formation during assembly within the bacterial periplasm.

To determine whether the bacterially expressed IgG retained the same binding avidity as those expressed in mammalian cell culture, an ELISA of serially diluted purified IgG expressed from both bacterial and mammalian cell cultures was carried out against Dengue-2 and epsilon toxin using 4G2 and ET149 respectively. A slight decrease in avidity was observed for both 4G2 and ET149 bacterially expressed IgG in comparison to mammalian cell culture derived IgG (Figure 5B). This could be due to an increased percentage of partially assembled IgG in the bacterially expressed antibodies as observed in the SDS-PAGE gels (Figure 5A). The partially assembled IgGs would have reduced avidity as there was only one antigen binding site present.

## Discussion

From our initial experiments, it was clear that IgG expression in *E. coli* laboratory strains using general expression protocols gave poor yields and highly degraded product and the use of the protease-deficient BL21 strain was unable to reduce degradation. Previous studies suggested that overload of the secretory system was responsible for the poor yield and we therefore undertook optimization of expression conditions to minimize load via reduction of translational levels. We have demonstrated in this paper on the basis of expression of five different antibodies that reduction of translational levels by using reduced inducer concentrations in combination with delayed induction improved yields of full-length IgG by both increasing the yield of wet cell mass, as well as in certain antibodies the unit yield (yield per unit wet cell mass), over standard induction conditions. This effect was observed only when high copy plasmids were used.

Interestingly, the effect of varying inducer concentration and induction OD on wet cell mass and unit yields was minimal when low copy plasmids were used. It may be that the low copy number results in a low baseline translation level which is not further reduced by induction OD or inducer concentration. While it does give a modest improvement in wet cell mass and in some antibodies tested, an increase in unit yields over standard conditions, we have found that generally the optimized high copy plasmid conditions perform better in terms of wet cell mass and unit yields. Whereas a later induction at higher OD appears to either have no effect or a mild positive effect on unit yields when high copy plasmids were used, particularly for PA38, PA64 or 4G2. Interestingly, for ET21 and ET149, it may be that these antibodies are stable enough to be properly folded even at higher translation rates and therefore reducing translation rates through induction at higher OD or reducing inducer concentration has no or a negative effect on unit yields. In particular, expression from the high copy plasmid induced at OD_600_ 1.0 with 20 ng/ml inducer, demonstrated optimal antibody yield for all antibodies tested either through direct increase of unit yield or through the increased cell number provided by the higher wet cell mass.

Purification of the cell lysate with Protein A was insufficient to give pure full-length IgG with bacterial proteins and heavy chain fragments co-purifying in the eluate and as a result subsequent Protein L purification was required. The inability of Protein A purification alone to give purified antibody, in contrast to previously reported findings, could be due to the extremely low yield of IgG resulting in non-specific binding of bacterial proteins to unoccupied Protein A binding sites. However, due to the large volume of lysate required to be processed for reasonable antibody yields, a sufficiently large Protein A column must be used for efficient antibody capture and hence having empty binding sites is an unavoidable consequence of the low yield of fully assembled IgG. Furthermore, this step is also required for the removal of the heavy chain degradation fragments.

The ability of our bacterial IgGs to bind both Protein A and Protein L indicates that the binding sites on the antibody are presented on the surface and suggests that the IgG is properly folded. Crucially, the purified 4G2 and ET149 antibodies were able to bind their respective antigens albeit with slightly reduced avidity as compared to mammalian cell expressed antibody and indicates that bacterially expressed IgG could substitute for mammalian derived IgG. *E. coli* expressed IgG1 has been found to bind the neonatal Fc receptor (FcRn) and have the same pharmacokinetic parameters in the circulatory system as mammalian cell expressed IgG2 and IgG4b, but was unable to bind complement (C1q) or FcγRI [8]. This suggests that *E. coli* derived antibodies lack effector functions as expected for aglycosylated antibodies. The inability of aglycosylated antibodies to bind Fcγ receptors has also been demonstrated in mammalian derived aglycosylated antibodies [16]. In addition, as reduction of translational levels appears to be beneficial to periplasmic expression in general, the strategies we have explored may also prove to be useful for improving the yields of other periplasmically expressed proteins.

## Materials and Methods

### Plasmid construction

The backbone of the expression plasmid was derived from pASK-IBA2 (IBA GmbH, Germany), which contains a tetracycline based promoter and an OmpA leader sequence for export into the periplasm. Onto this a bicistronic expression cassette was constructed where the light chain, the intercistronic ribosomal binding site, and the heavy chain variable region with the PelB leader sequence was cloned in as a complete construct from the phage display library via ApaL1 and BsmB1 cloning sites. The heavy chain constant region of human G1 was cloned in previously to be in frame with the variable heavy region. During the course of plasmid construction, deletion of a backbone restriction enzyme site situated within the origin of replication had created a reduced copy number plasmid in addition to the normal high copy number plasmid. For generation of the humanized 4G2 construct, the light chain and variable heavy chains was cloned in as separate constructs via ApaL1/AscI and Mfe/Xho1 restriction sites respectively. The four fully human antibodies were isolated by standard panning protocols against the respective purified antigen with the naïve human phage display library HX01 (Humanyx Pte Ltd, Singapore), and cloned into the bacterial IgG expression vector using ApaL1 and BsmB1.

### Optimization of expression with BL21(DE3)

The low copy number 4G2 expression plasmid was transformed into either HB2151 or BL21(DE3) *E. coli* and grown up in 2YT broth with 100 µg/ml carbenicillin. Induction was carried out at OD_600_ 1.0 with 1/10 normal inducer concentration (final concentration of 20 ng/ml). Periplasmic extraction was carried out by resuspending the pellet in 1/40 volume chilled periplasmic extraction buffer (100 mM Tris pH 8.0, 150 mM NaCl, 1 mM EDTA, 0.5 M sucrose, 1 mM PMSF, 1 µg/ml pepstatin A, 10 mM iodoacetamide), sonicating briefly on ice and then gently rotated at 10 rpm for 1 hr at 4°C. Cells were collected at 40000 g for 30 min at 4°C and the supernatant was retained.

### Small scale optimization of bacterial IgG expression conditions

Expression plasmids were transformed into *E. coli* strain HB2151 and grown up as a starter culture. 50 ml of 2YT broth with 100 µg/ml carbenicillin was inoculated with 1/50 volume of starter culture and grown at 30°C at 220 rpm. Induction was carried out as indicated and cultures transferred to room temperature and grown overnight at 120 rpm. The bacterial pellet was weighed and resuspended in chilled lysis buffer (20 mM sodium phosphate pH 7.4, 300 mM NaCl, 1 mM EDTA, 0.5 mM PMSF, 1 µg/ml pepstatin A, 10 mM iodoacetamide and Roche Complete protease inhibitor) at a ratio of 1 ml per 200 mg wet cell mass and sonicated on ice. Lysates were clarified by centrifugation at 16000 g for 30 min at 4°C. All cultures were grown up in non-baffled shake flasks and cytoplasmic extraction carried out as described above for all subsequent experiments.

### Large-scale expression and purification of bacterial IgG

Expression of 4G2 was carried out in 2×800 mL of 2YT media with 100 µg/ml carbenicillin in 2 L non-baffled shake flasks as described above with the following optimized conditions: high expression construct was used and induction was carried out at OD_600_ 1.0 using 20 ng/ml or 50 ng/ml final inducer concentration. The cell pellet was weighed and resuspended in lysis buffer at the same volume to wet cell mass ratio as above, sonicated and clarified by centrifugation at 20000 g for 30 min at 4°C. The cell lysate was passed through a 0.22 µm filter before purification on a 1 ml recombinant Protein A FF column (GE Healthcare, United Kingdom) fitted to a AKTAprime Plus or Explorer FPLC system (GE Healthcare, United Kingdom). Lysate was passed through the column at a flow rate of 0.5 ml/min, then the column washed with 30 ml 1× PBS before elution in 0.5 ml fractions with IgG elution buffer (Pierce, United States). Fractions were neutralized with 1/10 volume 1 M Tris pH 8.0. Peak fractions were pooled and buffer exchanged into 2 ml Protein L binding buffer (100 mM sodium phosphate buffer pH 7.2, 150 mM NaCl) with a Amicon 30000 MWCO concentrator (Millipore, United States) and then bound to 400 µl Protein L agarose bead 50% slurry (Pierce, United States) overnight at 4°C. The beads were washed with ten column volumes of 1× PBS in column and IgG eluted with IgG elution buffer (Pierce, United States) and neutralized as above. Eluate was buffer exchanged into 1× PBS/20% glycerol and concentrated down to 150 µl volume.

### Immunoblot & Protein quantitation

Samples taken during extraction and purification were resolved by SDS-PAGE with or without DTT and transblotted to a Hybond-C nitrocellulose membrane (GE Healthcare, United Kingdom). IgG was detected using HRPO labeled polyclonal anti-human Fc antibody (Roche, Switzerland) diluted 1∶2500 in 1× PBS with 0.05% Tween-20 and 5% skim milk or in combination with anti-human Kappa antibody (Roche, Switzerland) in the above diluent at 1∶5000 dilution each. Blots were washed eight times with 1× PBS with 0.05% Tween-20 and once with 1× PBS and then signal developed with SuperSignal West Pico Substrate (Pierce, United States). Samples with and without DTT were run on 10% and 8% SDS-polyacrylamide gels respectively. To measure protein concentration, 30 µl of Protein L purified IgG eluate was diluted in 1× PBS to 150 µl and measured by the Bradford method using the Coomassie Plus kit (Pierce, United States) according to manufacturer's protocol,

### Mammalian cell culture IgG expression and ELISA

Full-length 4G2 or ET149 IgG1 antibody was transiently expressed in 293T suspension culture. The antibody heavy and light chains were cloned into a biscistronic pCMV vector and transfected into cells using 293fectin (Invitrogen, United States) according to manufacturer's protocols. Expressed antibody was recovered from the culture supernatant and purified on Protein A (Pierce, United States).

ELISA was carried out by coating a Maxisorb 96-well ELISA plate (Nunc, Denmark) with 20 µg/ml of protein antigen or 50 µl of live DEN-2 virus (strain ST) in 1× PBS. For protein antigens, the plate was then washed twice with 1× PBS and blocked with 360 µl of blocking solution (4% skim milk in 1× PBS) for 2 hrs at room temperature. For dengue ELISAs virus was captured using mouse 4G2 monoclonal coated at 5 µg/ml and blocked as above prior to the addition of virus. Virus was allowed to bind for 1 hr at room temperature and excess virus removed with two washes with 1× PBS/0.05% Tween-20.

100 µl of antibody (diluted at a 1∶2 ratio from 5 µg/ml to 0.076 µg/ml) or clarified cell lysate (at dilutions from 1∶2 to 1∶64) was then added in blocking solution for 1 hr followed by secondary antibody, HRPO labeled polyclonal anti-human IgG Fc antibody (Roche, Switzerland) at 1∶5000 dilution, also in blocking solution, for another 1 hr. TMB (Pierce, United States) was added to develop color and stopped with 2 M sulfuric acid. All steps except the blocking step were carried out in 100 µl volumes and at room temperature except for ELISAs involving dengue virus which were done at 50 µl volumes. The ELISA plate was washed four times with 1× PBS/0.05% Tween-20 between each step after blocking except prior to addition of TMB in which the last wash was with 1× PBS instead. For ELISAs involving dengue virus only two 1× PBS/0.05% Tween-20 washes were carried out except for the last prior to addition of TMB where an additional 1× PBS wash was done.

## References

[1] Chadd HE, Chamow SM (2001). Therapeutic antibody expression technology.. Curr Opin Biotechnol.

[2] Jefferis R, Lund J, Pound JD (1998). IgG-Fc-mediated effector functions: molecular definition of interaction sites for effector ligands and the role of glycosylation.. Immunol Rev.

[3] Tao MH, Morrison SL (1989). Studies of aglycosylated chimeric mouse-human IgG. Role of carbohydrate in the structure and effector functions mediated by the human IgG constant region.. J Immunol.

[4] Presta LG (2006). Engineering of therapeutic antibodies to minimize immunogenicity and optimize function.. Adv Drug Deliv Rev.

[5] Jefferis R (2005). Glycosylation of recombinant antibody therapeutics.. Biotechnol Prog.

[6] Wright A, Morrison SL (1994). Effect of altered CH2-associated carbohydrate structure on the functional properties and in vivo fate of chimeric mouse-human immunoglobulin G1.. J Exp Med.

[7] Wright A, Morrison SL (1998). Effect of C2-associated carbohydrate structure on Ig effector function: studies with chimeric mouse-human IgG1 antibodies in glycosylation mutants of Chinese hamster ovary cells.. J Immunol.

[8] Simmons LC, Reilly D, Klimowski L, Raju TS, Meng G (2002). Expression of full-length immunoglobulins in Escherichia coli: rapid and efficient production of aglycosylated antibodies.. J Immunol Methods.

[9] Mazor Y, Van Blarcom T, Mabry R, Iverson BL, Georgiou G (2007). Isolation of engineered, full-length antibodies from libraries expressed in Escherichia coli.. Nat Biotechnol.

[10] Venturi M, Seifert C, Hunte C (2002). High level production of functional antibody Fab fragments in an oxidizing bacterial cytoplasm.. J Mol Biol.

[11] Levy R, Weiss R, Chen G, Iverson BL, Georgiou G (2001). Production of correctly folded Fab antibody fragment in the cytoplasm of Escherichia coli trxB gor mutants via the coexpression of molecular chaperones.. Protein Expr Purif.

[12] Simmons LC, Yansura DG (1996). Translational level is a critical factor for the secretion of heterologous proteins in Escherichia coli.. Nat Biotechnol.

[13] Ehrt S, Guo XV, Hickey CM, Ryou M, Monteleone M (2005). Controlling gene expression in mycobacteria with anhydrotetracycline and Tet repressor.. Nucleic Acids Res.

[14] Lutz R, Bujard H (1997). Independent and tight regulation of transcriptional units in Escherichia coli via the LacR/O, the TetR/O and AraC/I1-I2 regulatory elements.. Nucleic Acids Res.

[15] Skerra A (1994). Use of the tetracycline promoter for the tightly regulated production of a murine antibody fragment in Escherichia coli.. Gene.

[16] Walker MR, Lund J, Thompson KM, Jefferis R (1989). Aglycosylation of human IgG1 and IgG3 monoclonal antibodies can eliminate recognition by human cells expressing Fc gamma RI and/or Fc gamma RII receptors.. Biochem J.

